# Advancements in left ventricular assist devices to prevent pump thrombosis and blood coagulopathy

**DOI:** 10.1111/joa.13675

**Published:** 2022-04-20

**Authors:** Grainne Malone, Gerges Abdelsayed, Fianait Bligh, Fatma Al Qattan, Saifullah Syed, Prateepan Varatharajullu, Augustin Msellati, Daniela Mwipatayi, Maimoona Azhar, Andrew Malone, Saulat H. Fatimi, Claire Conway, Aamir Hameed

**Affiliations:** ^1^ Tissue Engineering Research Group (TERG) Department of Anatomy and Regenerative Medicine, RCSI University of Medicine and Health Sciences, Dublin 2 Dublin Ireland; ^2^ School of Medicine RCSI University of Medicine and Health Sciences, Dublin 2 Dublin Ireland; ^3^ School of Pharmacy and Biomolecular Sciences RCSI University of Medicine and Health Sciences, Dublin 2 Dublin Ireland; ^4^ Department of Surgery St. Vincent's University Hospital, Dublin 4 Dublin Ireland; ^5^ Department of Cardiothoracic Surgery Aga Khan University Hospital Karachi Pakistan; ^6^ Trinity Centre for Biomedical Engineering (TCBE) Trinity College Dublin (TCD) Dublin Ireland

**Keywords:** blood coagulopathy, heart failure, left ventricular assist devices, mechanical circulatory support, pump thrombosis, thrombus

## Abstract

Mechanical circulatory support (MCS) devices, such as left ventricular assist devices (LVADs) are very useful in improving outcomes in patients with advanced‐stage heart failure. Despite recent advances in LVAD development, pump thrombosis is one of the most severe adverse events caused by LVADs. The contact of blood with artificial materials of LVAD pumps and cannulas triggers the coagulation cascade. Heat spots, for example, produced by mechanical bearings are often subjected to thrombus build‐up when low‐flow situations impair washout and thus the necessary cooling does not happen. The formation of thrombus in an LVAD may compromise its function, causing a drop in flow and pumping power leading to failure of the LVAD, if left unattended. If a clot becomes dislodged and circulates in the bloodstream, it may disturb the flow or occlude the blood vessels in vital organs and cause internal damage that could be fatal, for example, ischemic stroke. That is why patients with LVADs are on anti‐coagulant medication. However, the anti‐coagulants can cause a set of issues for the patient—an example of gastrointestinal (GI) bleeding is given in illustration. On account of this, these devices are only used as a last resort in clinical practice. It is, therefore, necessary to develop devices with better mechanics of blood flow, performance and hemocompatibility. This paper discusses the development of LVADs through landmark clinical trials in detail and describes the evolution of device design to reduce the risk of pump thrombosis and achieve better hemocompatibility. Whilst driveline infection, right heart failure and arrhythmias have been recognised as LVAD‐related complications, this paper focuses on complications related to pump thrombosis, especially blood coagulopathy in detail and potential strategies to mitigate this complication. Furthermore, it also discusses the LVAD implantation techniques and their anatomical challenges.

## Graphical abstract

Left ventricular assist device (LVAD)‐related blood coagulopathy: Pump thrombosis is one of the most severe adverse events caused by LVADs. The contact of blood with artificial materials of LVAD pumps and cannulas triggers the coagulation cascade.

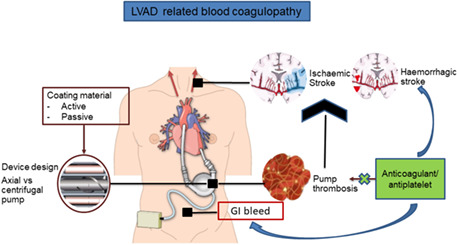

## 1 INTRODUCTION

Heart failure (HF) is a complex clinical syndrome that is identified by the reduced ability of the heart to fill and/or pump blood (Coronel et al., 2001; Savarese & Lund, 2017; Tan et al., 2010). From a physiological point of view, HF may be defined as an inadequate cardiac output to meet the body's metabolic demands, or an adequate cardiac output secondary to compensatory activation, which tends to manifest as increased left ventricular filling pressures (Rosalia et al., 2021; Savarese & Lund, 2017).

HF is a leading cause of death worldwide, constituting a major clinical and public health problem, with a prevalence in excess of 26 million people (Savarese & Lund, 2017). It is estimated that HF currently affects 6.3 million individuals in the United States, with a projected 46% increase to over 9 million people by the year 2030 (Levine & Gass, 2019). This trajectory is due to a number of factors, for example, an ageing population, the pervasiveness of HF risk factors, such as hypertension, coronary artery disease and obesity. The economic burden created by HF is also significant. In the US alone, the annual total direct medical costs for patients with HF currently stand at $39 billion and are expected to increase to $53 billion by the year 2030 (Rosalia et al., 2021).

Developments in the area of HF pharmacotherapy, especially localised delivery of various therapeutics, for example, proteins, drugs and stem cells to the heart, targeting various pathophysiological aspects of the HF are substantial (Hameed et al., 2019; Kathryn et al., 2021). However, they may not be an option for advanced‐stage HF patients. Advanced or medically refractory HF represents the end‐stage form of heart disease, and carries a life expectancy of less than 2 years (Combes, 2017; Kennelly et al., 2021). While heart transplantation remains the ‘gold standard’ for definitive treatment, this treatment modality is significantly limited due to the severe imbalance between donor heart supply and demand (Cai et al., 2017; Siân Pincott & Burch, 2011). In the US alone, approximately 3200 orthotropic heart transplantations are performed annually, despite an estimated 250,000 patients with New York Heart Association (NYHA) class IIIB or IV symptoms that are refractory to medical treatment, making such patients potential candidates for heart transplantation (Ahmed, 2007; Alraies & Eckman, 2014). Furthermore, many patients deteriorate while awaiting a donor heart to become available, culminating in irreversible multi‐organ failure, rendering them an unsuitable candidate for a transplant. This discrepancy, along with the increasing incidence of HF has prompted efforts towards the development of alternative approaches, namely mechanical circulatory support (MCS) devices to minimise the mortality rates.

While each MCS device has its own unique characteristics, most systems can be classified into two categories: Ventricular assist devices (VADs) and total artificial hearts (TAH). VADs are mechanical pumps designed to augment the function of the failing heart in order to re‐establish normal hemodynamic and end‐organ blood flow and may provide support to either left (LVAD), right (RVAD) or both ventricles (BiVAD) (Cai et al., 2017; Hosseinipour et al., 2017). TAHs, on the other hand, replace a portion or the entire heart, similar to a prosthesis elsewhere in the human body. Since the first implantation in 1966, LVADs have become an established worldwide treatment modality for HF patients (Chair et al., 2016; DeBakey, 1971; Hosseinipour et al., 2017; Prinzing et al., 2016). These devices may be employed as a ‘bridge to transplantation (BTT)’, whereby they provide mechanical support to the heart for patients awaiting transplantation, a ‘bridge to recovery (BTR), where mechanical support provided by them allows for sufficient myocardial recovery following which the device can be removed, or as a ‘destination therapy (DT)’, where mechanical support is provided for patients who are deemed ineligible for transplantation due to age, malignancy or other co‐morbidities (Chair et al., 2016; Holley et al., 2014; Lahpor, 2009; Rodriguez et al., 2013).

Hence, MCS devices like LVADs are very useful in improving outcomes in patients with advanced‐stage heart disease, however, their use is limited by not only their mechanical features, for example, large size and a lack of durability, but also because of potential complications (Bartoli & Dowling, 2019). Despite recent advances in LVAD development, pump thrombosis is one of the most severe adverse events caused by LVADs. All VAD systems activate the blood coagulation system via several mechanisms. The blood contact with the artificial materials of VAD pumps and cannulas triggers the coagulation cascade as well as high shear stress due to high flow velocities, small gaps or high‐speed moving parts, such as the impeller of the rotary blood pumps (Potapov et al., 2017; van Oeveren, 1995). Areas of flow stagnation represent preferred thrombus developing sites as well as areas with recirculation vortices within the pump. Heat spots, for example, produced by mechanical bearings are often subjected to thrombus build‐up when low‐flow situations impair washout and thus the necessary cooling does not happen (Potapov et al., 2017). The formation of thrombosis in an LVAD may compromise its function, causing a drop in flow and pumping power leading to failure of the LVAD, if left unattended (Singhvi & Trachtenberg, 2019). If the clot becomes dislodged and circulates in the bloodstream, it may disturb the flow or occlude the blood vessels in vital organs and cause internal damage that could be fatal. That is why patients with LVADs are on anticoagulant medication.

On account of this, these devices are only used as a last resort in clinical practice. It is, therefore, necessary to develop devices with better mechanisms of blood flow, performance and hemocompatibility. This paper describes the development of LVADs through landmark clinical trials in detail and describes the evolution of device design to reduce the risk of pump thrombosis and achieve better hemocompatibility. Whilst driveline infection, right heart failure and arrhythmias have been recognised as LVAD‐related complications, this paper focuses on complications related to pump thrombosis, especially blood coagulopathy in detail and potential strategies to mitigate this complication. Furthermore, it also discusses the LVAD implantation techniques and their anatomical challenges.

## 2 LEFT VENTRICULAR ASSIST DEVICE DESIGN

Since its inception in 1962, all generations of implantable LVAD technology have the same three essential components: an inflow cannula in the left ventricle, a pump and an outflow graft, which delivers blood to the arterial circulation through the aorta. The pump is, in turn, powered via a tunnelled driveline that connects to an external controller and power source (Levine & Gass, 2019).

### 2.1 First‐generation LVADs

First‐generation LVADs, otherwise known as volume displacement pumps, were designed to replicate the native cardiopulmonary circulation by driving pulsatile blood flow via a pulse generator (Lahpor, 2009; Levine & Gass, 2019). These pumps incorporated unidirectional inlet and outlet valves, along with a moveable diaphragm that could be displaced to periodically/cyclically increase the internal pump volume to facilitate blood filling during the diastolic phase while subsequently compressing in order to eject blood during the systolic phase (Cai et al., 2017; Levine & Gass, 2019). These pumps were connected to an external driver, either a pneumatic or an electrical pump, which provided alternating pressures required to generate the characteristic pulsatile flow (Cai et al., 2017). Examples of first‐generation LVADs include the HeartMate XVE (Thoratec Inc.), Abiomed AB5000 (Abiomed Inc.), Berlin Heart EXCOR (Berlin Heart Inc.).

#### 2.1.1 REMATCH trial—Demonstrated clinical efficacy of LVADs

The landmark clinical trial that launched the LVAD into widespread clinical use was the Randomized Evaluation of Mechanical Assistance for the Treatment of Congestive Heart Failure (REMATCH) trial, published in the New England Journal of Medicine in 2001 (Levine & Gass, 2019; Rose et al., 2001). The primary objective of the REMATCH trial was to evaluate the reliability and effectiveness of LVADs in chronic end‐stage heart failure patients (class IV, as per New York Heart Association scale) from May 1998 to July 2001 (Chair et al., 2016; Rose et al., 2001). A total of 129 patients deemed ineligible for cardiac transplantation were randomised to either LVAD implantation with the HeartMate XVE device (68 patients) or optimal medical therapy (61 patients).

The trial demonstrated a dramatic 48% reduction in all‐cause mortality in the LVAD group compared to the medical‐therapy group (relative risk, 0.52; 95% confidence interval 0.34–0.78; *p* = 0.001). The rates of survival were superior in the device group at 1 year (52% vs. 25%, *p* = 0.002) and 2 years (23% vs. 8%, *p* = 0.09), respectively, while compared to the medical therapy group, a meaningful improvement in quality of life was observed in the device group. On the basis of these results, the HeartMate XVE received FDA approval, and LVADs were deemed an acceptable alternative therapy for chronic HF patients (Chair et al., 2016). Despite this substantial survival benefit, the frequency of serious adverse events in the device group was 2.35 times that in the medical therapy group.

First‐generation LVADs succumbed to a limited lifetime and low reliability due to extensive mechanical wear and failure on repeatedly moving parts, such as diaphragms, valves and bearings (Cai et al., 2017; Levine & Gass, 2019). Due to their large volume, extensive surgical dissection was necessary to create the pump pocket, which posed a high risk for driveline infection, bleeding and thromboembolic complications (Birks, 2010; Lahpor, 2009). Consequently, initial VADs were limited to recipients who had a body surface area of at least 1.5 m^2^ to accommodate the device, thereby restricting women and adolescents from device candidacy (Cai et al., 2017). With the risks of these extensive device complications, this pulsatile pump design was not a preferable option for long‐term support.

### 2.2 Second‐generation LVADs

**FIGURE 1:**
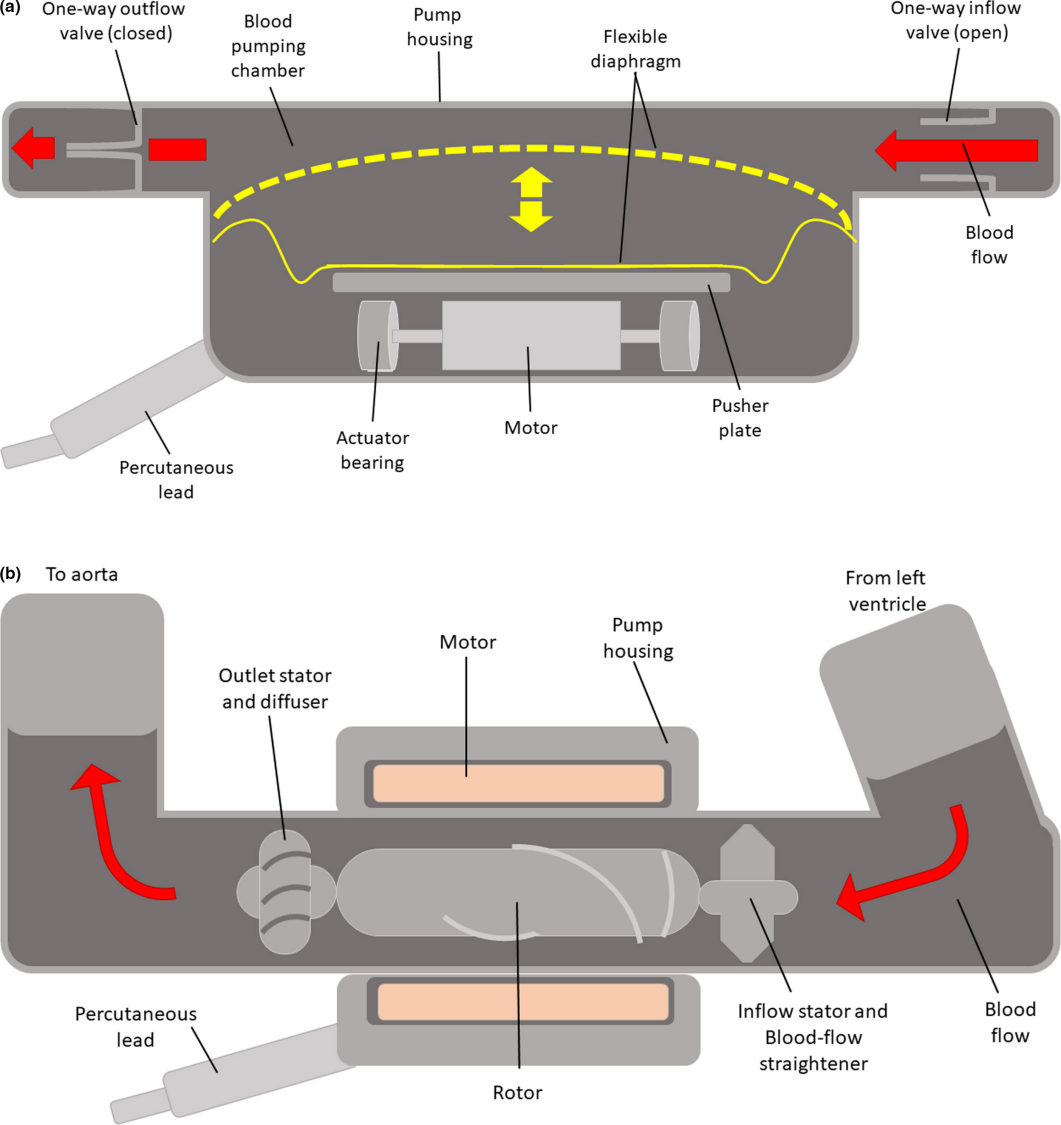
(a) Pulsatile flow, (b) continuous flow—left ventricular assist device (adapted from Slaughter et al. (2009)

Following the REMATCH trial, second‐generation LVADs were developed with a focus on miniaturisation, improved efficiency and greater mechanical reliability, in order to combat the adverse events associated with first‐generation devices (Cai et al., 2017; Levine & Gass, 2019). This paradigm shift saw the introduction of rotary pumps, which operated via continuous blood flow as opposed to the characteristic pulsatile flow generated by their pulsatile predecessor(s), as shown in Figure 1 (Levine & Gass, 2019; Slaughter et al., 2009).

The rotary heart pump design may be classified into two categories based on the pump's structure: axial flow pumps and centrifugal pumps. In terms of pump design theory, axial flow pumps typically generate higher flows at lower pressure rises, while centrifugal pumps are capable of producing higher pressures at lower flows (Hosseinipour et al., 2017; Shah et al., 2017). All second‐generation pumps are based on axial flow pump technology, which consists of a single rotating element, known as the impeller, suspended by mechanical contact bearings within a stationary housing unit, which directs continuous flow linearly along the axis of rotation (Levine & Gass, 2019).

Much of the success of second‐generation LVADs stems from the simplicity of their design. Pump miniaturisation was achieved by eliminating extraneous components such as valves and compliance chamber (Cai et al., 2017; Chair et al., 2016). This reduction in size is highly advantageous as it increases the versatility of the patient cohort, while also facilitating less invasive surgical procedures, thereby improving patient comfort and decreasing the risk of infection. Furthermore, by relying on a single moving part, the continuous flow second‐generation LVADs possess greater durability and enhanced mechanical reliability due to lower mechanical wear and tear on moving parts (Levine & Gass, 2019). Examples of second generation devices include the HeartMate™ II (Thoratec Inc.), the Jarvik 2000® (Jarvik Heart, Inc.) and the DeBakey® LVAD (MicroMed Technology, Inc.). To date, the HeartMate II is the most successful second‐generation device, with over 2500 implants worldwide (Garbade et al., 2011).

#### 2.2.1 HeartMate II trial

One of the initial trials that launched the era of second‐generation LVAD devices was the ‘Advanced Heart Failure Treated with Continuous‐Flow Left Ventricular Assist Device study, published in 2009 by Slaughter et al (Slaughter et al., 2009). In this randomised, multicentre trial, a total of 200 patients with end‐stage HF were randomly assigned to undergo implantation of a continuous flow HeartMate LVAD (*n* = 134), or its pulsatile flow predecessor, the HeartMate XVE (*n* = 66). The primary composite endpoint was, at 2 years, survival free of disabling stroke, or reoperation to repair or replace the pump (Slaughter et al., 2009).

At 2 years, the trial showed that the primary composite endpoint was achieved in more patients with the continuous flow HeartMate II device, compared to the pulsatile flow Heartmate XVE device (46% vs. 11%, respectively, *p* < 0.001), with rates of survival alone also being superior in the HeartMate II group (58% vs. 24%, *p* = 0.008) (Slaughter et al., 2009). Moreover, the frequency of adverse events, including infection (device‐ and non‐device‐related), right heart failure and cardiac arrhythmia, and the need for device replacements were significantly lower in the continuous flow group, while the quality of life and functional capacity improved significantly in both groups compared to baseline (Slaughter et al., 2009). Interestingly, the incidence of stroke did not differ significantly between the continuous‐f low group and the pulsatile‐flow group (0.13 vs. 0.22 events per patient‐year, respectively, *p* = 0.21) (Slaughter et al., 2009).

Despite overcoming many of the shortcomings of their first‐generation counterparts, second‐generation LVADs succumb to several limitations. The impeller, which rotates at high speeds of 10,000–20,000 rpm, is culpable for generating excessive amounts of shear stresses, which over time may lead to serious adverse events such as hemolysis, thrombus formation and device failure, restricting the efficacy of the pump (Nguyen et al., 2016). In order to mitigate the risk of thrombosis, patients with second‐generation LVADs often require high doses of anticoagulants, which increases their risks of bleeding. Regurgitation is also possible due to the lack of valves to prevent backflow.

The clinical manifestation of pump thrombosis took the forefront for HeartMate II LVADs in 2014 (Levine & Gass, 2019). While initial trials demonstrated low rates of pump thrombosis, an apparent increase in the rate of pump thrombosis at the Cleveland Clinic led to the pooling of data with two other major centres, Barnes‐Jewish Hospital (Washington University School of Medicine) and Duke University Medical Centre, for the landmark report by Starling et al (Starling et al., 2014). From March 2011, Starling et al reported an abrupt increase in the incidence of pump thrombosis, with rates of pump thrombosis increases from 2.2% to 8.4% over a 3‐month period (Starling et al., 2014). This increased rate of pump thrombosis brought with it a substantial increase in morbidity and mortality. At 180‐days post‐implantation, patient mortality was more than twofold higher than in patients without thrombosis (35.6% vs. 16.8%) (Starling et al., 2014). These results led to significant changes in clinical practice protocols, highlighted by the PRE‐VENT (Prevention of HeartMate II Pump Thrombosis through Clinical Management) trial, which was designed with the aim of lowering the rates of pump thrombosis in HeartMate II device (Maltais et al., 2017). By adhering to this pre‐determined set of surgical and subsequent clinical management practices, lower rates of early pump thrombosis with HeartMate II may be achieved. These results emphasise the importance of LVAD implantation techniques, adequate anti‐coagulation treatment including post‐operative heparin bridging, and optimal speed management (<9000 RPMs) (Cai et al., 2017; Levine & Gass, 2019; Maltais et al., 2017).

### 2.3 Third‐generation LVADs

The transition from second‐ to third‐generation rotary blood pumps is defined by the introduction of magnetic and/or hydrodynamic bearing technology, whereby the impeller is suspended entirely within the blood field (Hosseinipour et al., 2017; Levine & Gass, 2019). The elimination of all mechanical contacts between the impeller and the drive mechanism enables friction‐free rotation of the impeller with minimal heat generation, leading to increased mechanical durability necessary for long‐term mechanical circulatory support, with estimated reliability of 10 years (Levine & Gass, 2019; Prinzing et al., 2016). Moreover, the elimination of mechanical contact bearings allows for a more complete washing of the blood‐contacting surfaces, preventing the incidence of stasis and recirculation vortices in pro‐thrombotic sites, thereby enhancing pump biocompatibility (Levine & Gass, 2019).

In contrast to second‐generation LVADs, which consist of axial‐flow pumps, third‐generation LVADs operate exclusively via a centrifugal pump, where the rotating impeller propels blood perpendicular to the axis of rotation via centrifugal force (Hosseinipour et al., 2017; Moazami et al., 2013). Centrifugal flow pumps offer certain advantages over axial flow pumps, including lower rotational speeds and greater afterload sensitivity, that is, demonstrate more significant changes in flow for a given change in pressure. For this reason, centrifugal pumps can decrease inlet suction force at times of low flow; reducing the risk of ventricular arrhythmias (Cai et al., 2017; Moazami et al., 2013).

With the introduction of third‐generation LVADs, another significant size reduction could be achieved. Unlike first‐ and second‐generation devices, which are positioned in a sub‐diaphragmatic pocket, third‐generation pumps sit entirely within the pericardial space, which reduces the risks of infection and surgical trauma (Levine & Gass, 2019). Examples of third‐generation devices are the VentrAssist™ (Ventracor Ltd., Sydney, Australia), DuraHeart™ (Terumo, Inc.), the HVAD (HeartWare Corp.) and the EVAHEART™ LVAS (Sun Medical Technology Research Corporation).

#### 2.3.1 ADVANCE trial

The ADVANCE trial was the initial trial that launched the era of third‐generation centrifugal flow pumps (Cai et al., 2017; Levine & Gass, 2019). In this study, patients with end‐stage HF who were deemed eligible for cardiac transplantation were implanted with the HVAD device (*n* = 140) and compared to a control cohort (*n* = 499) from the Interagency Registry for Mechanically Assisted Circulatory Support (INTERMACS) registry who received a commercially available LVAD, the second generation HeartMate II as a BTT (Levine & Gass, 2019; Najjar et al., 2014).

The trial confirmed HVAD non‐inferiority to the control device, with 90.7% success defined by transplant, recovery explant or survival with HVAD at six months in the study arm versus 90.1% (*p* < 0.001) in the control group (Najjar et al., 2014; Nguyen et al., 2016). Regression analysis at 30, 60, 120 and 360 days post‐enrolment consistently showed equivalent survival in the HVAD and control groups (Cai et al., 2017; Najjar et al., 2014). Secondary endpoints of functional capacity and quality‐of‐life scores improved markedly, with a favourable adverse event profile, similar to those obtained with cardiac transplantation (Najjar et al., 2014). The results of this trial led to FDA approval of HVAD for BTT in 2012, along with a subsequent Continued Access Protocol (CAP) trial for the BTT indication (Najjar et al., 2014).

### 2.4 Second‐generation vs third‐generation LVADs—Endurance trial

The first head‐to‐head trial between second‐and third‐generation LVADs was the ENDURANCE trial, published in 2017 (Levine & Gass, 2019). Here, 446 advanced heart failure patients were randomly assigned, in a 2:1 ratio, to the investigational centrifugal flow HVAD or the control HeartMate II, an axial flow device (Levine & Gass, 2019; Milano et al., 2018; Rogers et al., 2017).

The HVAD demonstrated non‐inferiority to the control with respect to overall survival 2 years post‐enrollment (55.0% vs. 57.4%, *p* = 0.006) (Rogers et al., 2017). Quality of life and functional capacity improved to a similar degree in the two cohorts. Interestingly, fewer patients in the study arm experienced device malfunction or device failure requiring urgent explantation or transplantation than did patients who received the study device (16.2% vs. 8.8%). As a result, the rate of pump replacement for the HVAD was lower than for HeartMate II (7.8% vs. 13.4%, *p* = 0.06). While it was anticipated that the bearing‐less design of the HVAD would bring lower rates of pump thrombosis, no significant difference was apparent between the two cohorts. However, rates of stroke were alarmingly higher in the HVAD group (29.7% vs. 12.1%, *p* < 0.001), both for ischemic stroke (17.6% vs. 8.1%, *p* = 0.007) and hemorrhagic stroke (14.9% vs. 4.0%, *p* < 0.001) (Cai et al., 2017; Levine & Gass, 2019; Rogers et al., 2017). Post hoc analyses revealed increased mean arterial blood pressure (<90 mmHg) as a significant independent risk factor for stroke (Rogers et al., 2017).

These results led to the follow‐up Endurance Supplemental trial, a prospective, multicentre study evaluation that aimed to evaluate the impact of blood pressure management on stroke rates in patients receiving the HeartWare HVAD System (Levine & Gass, 2019). The trial confirmed that by adhering to a blood pressure management protocol, maintaining mean arterial pressure – MAP <90 mmHg, the HVAD demonstrated superiority in terms of freedom from death, disabling stroke and device replacement or urgent transplantation (76.1% vs. 66.9%, *p* = 0.04), which was the primary endpoint of the original ENDURANCE trial. The incidence of stroke in HVAD subjects was reduced by 24.2% in ENDURANCE Supplemental compared with ENDURANCE (*p* = 0.10), with hemorrhagic stroke being reduced by 50.5% (10.5% vs. 5.2%, *p* = 0.02) (Levine & Gass, 2019; Milano et al., 2018).

### 2.5 Centrifugal vs axial flow pump—MOMENTUM 3 study

In the Multicenter Study of MagLev Technology in Patients Undergoing Mechanical Circulatory Support Therapy with HeartMate 3 (MOMENTUM 3), the HeartMate 3 centrifugal‐flow left ventricular assist device was compared with the HeartMate II axial‐flow device in terms of safety and efficacy (Levine & Gass, 2019, Tamez et al., 2017, Mehra et al., 2018). In order to determine the safety and efficacy of the Heartmate 3 device, 1020 patients with advanced‐stage heart failure were randomised in a 1:1 ratio and implanted with either the HeartMate 3 (*n* = 515) or HeartMate II (*n* = 505) pump, irrespective of the intended goal of use (bridge to transplantation or destination therapy) (Mehra et al., 2018).

At 2 years, the HeartMate 3 proved superior to the HeartMate II with respect to the primary endpoint, with 76.9% of patients in the HeartMate 3 group achieving survival free of death, disabling stroke or reoperation to remove or replace a malfunctioning device, as compared to 64.8% in the latter (*p* < 0.001 for superiority) (Cai et al., 2017; Levine & Gass, 2019; Mehra et al., 2018). The rate of pump replacement was significantly lower in the centrifugal pump group than in the axial flow pump group (2.3% vs. 11.3%, *p* < 0.001), which was primarily driven by a 12.5% absolute reduction in pump thrombosis rates with the HeartMate 3, (1.4% vs. 13.9%, *p* < 0.001) (Mehra et al., 2018). This virtual elimination of pump thrombosis may be largely attributed to the technological advancements and improved design of the third‐generation centrifugal pump over its axial predecessor (Levine & Gass, 2019). With large gaps within the blood flow path and a contactless impeller design, shear stresses within the pump are reduced, while the artificial pulse ensures a thorough washing of blood‐contacting surfaces to reduce the risk of blood stasis (Levine & Gass, 2019). Moreover, the rates of adverse events, including stroke of any kind, major bleeding and gastrointestinal haemorrhage, were consistently lower in the centrifugal‐flow pump group than in the axial‐flow pump group (Mehra et al., 2018).

## 3 ADVERSE EVENTS—LVAD‐RELATED BLOOD COAGULOPATHY

**FIGURE 2:**
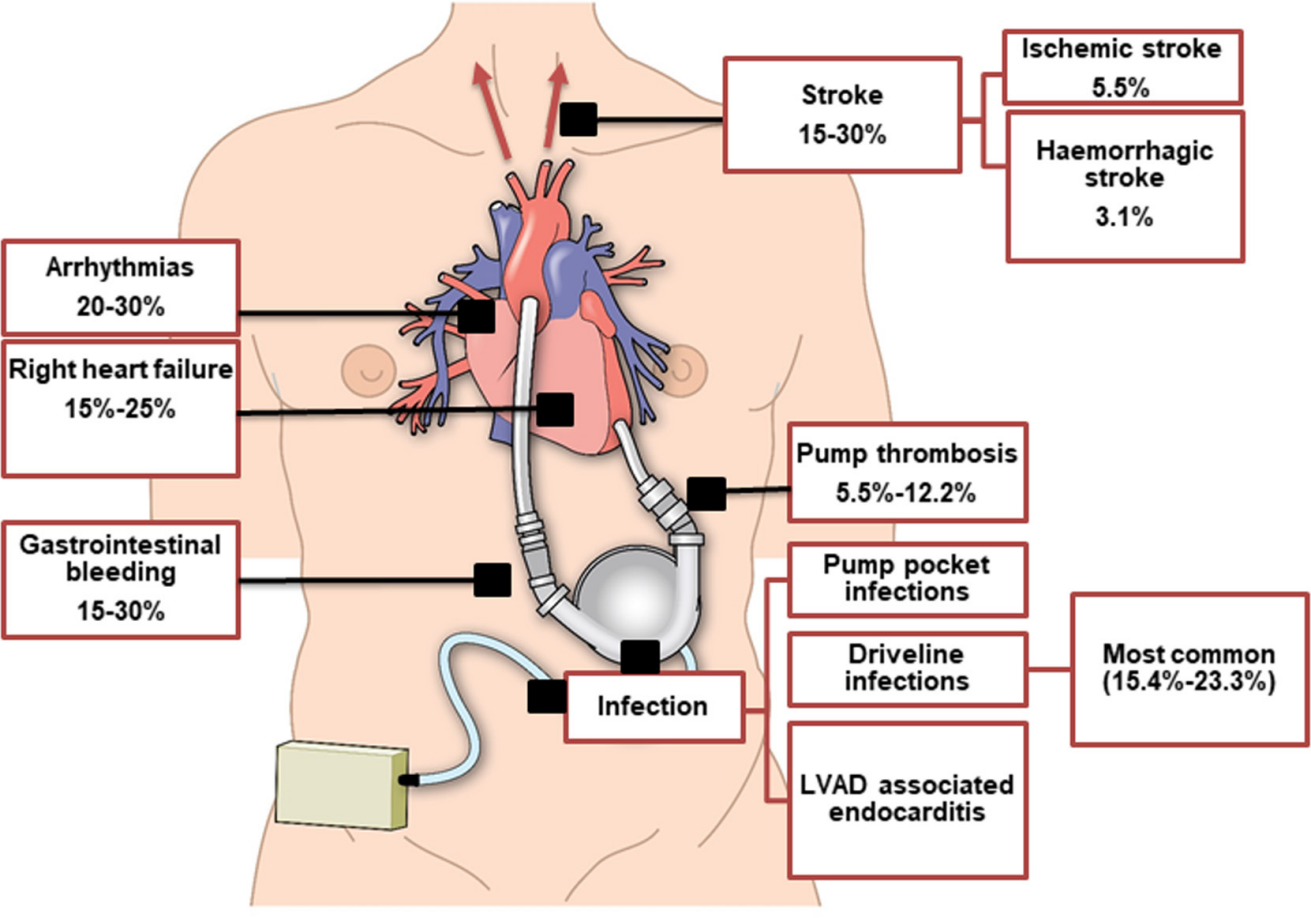
LVAD‐related complications and their incidence rates. Arrhythmias (Ozcan & Deshmukh, 2020), right heart failure (Han et al., 2018; Kilic et al., 2015), gastrointestinal bleeding (Goldstein et al., 2015; Stulak et al., 2014), stroke (Han et al., 2018; Rogers et al., 2017), pump thrombosis (Cook et al., 2017), driveline infections (Rogers et al., 2017). LVAD, left ventricular assist device

Despite advances in the LVAD field and their great benefit, their use is associated with complications, for example, pump thrombosis, stroke, bleeding, infection, right heart failure, aortic insufficiency and arrhythmias (Cook et al., 2017; Han et al., 2018; Kilic et al., 2015; Ozcan & Deshmukh, 2020). Figure 2 shows the LVAD‐related complications and their incidence rates. Early detection and management of these complications are crucial to improve the outcome.

### 3.1 Pump thrombosis

Pump thrombosis is a relatively frequent complication with a reported incidence of 5.5%–12.2% and is associated with significant morbidity (Cook et al., 2017). Numerous factors contribute towards this complication. They can be device‐related, including outflow graft kinking, extrinsic compression, low pump speed and local heat generated by the pump; patient‐related owing to pro‐thrombotic conditions including elevated blood pressure, infection, congestive heart failure and hypercoagulable states; and management‐related factors resulting from subtherapeutic and inadequate anticoagulation (Cook et al., 2017; Kilic et al., 2015). Patients with bleeding events such as gastrointestinal bleeding are predisposed to a higher risk of thrombosis owing to the need of decreasing or discontinuing anticoagulation (Kilic et al., 2015).

Pump thrombosis is associated with increased morbidity and mortality if not promptly and effectively managed. Elevated lactate dehydrogenase can occur up to 3 months prior to pump thrombosis which can be monitored routinely as a biomarker for haemolysis and pump thrombosis, which may help in early detection and thus management (Kilic et al., 2015; Starling et al., 2014).

#### 3.1.1 Hydrodynamic effects of thrombus material in an LVAD

A considerable size thrombotic material may cause a narrowing of the flow path in one or more sections of the LVAD, resulting in the reduction of LVAD's pumping capacity, due to elevated flow resistance (Potapov et al., 2017). Clinically, this would lead to low pump flow, thereby reducing the unloading of the left ventricle, which will manifest as signs of heart failure in the patient. Symptoms such as dyspnea, weakness or dizziness may occur, and ultimately, patient may go into cardiogenic shock (Potapov et al., 2017).

##### Mechanical effects of thrombus material in an LVAD

If thrombus material becomes lodged in the space between the rotating impeller and the stationary pump housing (Figure 1), energy will be lost due to friction (Potapov et al., 2017). To overcome this loss in energy, more power is required in order to maintain the pre‐set rotational speed. As a result, pump thrombosis may lead to false high flow readings. Most rotary blood pumps estimate the flow generated by the pump from its power consumption reading. To complicate the situation, the combined effect of low flow and increased power may compensate for each other, resulting in unsuspicious flow readings despite thrombotic material causing both flow reduction and power increase (Potapov et al., 2017). This could be fatal, if it goes undetected over a period of time.

##### Effects on the corpuscular blood components

Thrombi which are located at highly sensitive areas in LVADs, such as regions of turbulent flow velocities or high shear stress often increases the shear rate and thus haemolysis (Potapov et al., 2017). Haemolysis may be used as an indicator of pump thrombosis, even in cases of minimal alterations in flow or power consumption. As a general rule, the presence of haemolysis mandates hospital admission and further diagnostic testing. Haemolysis leads to an elevated risk of LVAD‐related adverse events, resulting in increased morbidity and mortality (Potapov et al., 2017).

##### Positioning of LVAD inflow and outflow graft cannulas

According to the 2019 European Association for Cardiothoracic Surgery (EACTS) Expert Consensus, the placement of inflow cannula is preferred in the LV apex and anterior wall (Potapov et al., 2019). Positioning of inflow cannula is important in preventing the cannula from penetrating too deep into the LV chamber. Such error can impact the LV hemodynamics and unloading, which may increase the risk of pump thrombosis (Chivukula et al., 2018; Chivukula et al., 2019). The Standard approach for placing the inflow cannula involves a full median sternotomy for the LV apex inflow cannula and ascending aorta outflow graft (Potapov et al., 2019). Compared to the standard approach, a sternotomy‐sparing technique, for example, left lateral thoracotomy, allows for direct access to the apex, which promotes better inflow cannula placement. This results in better pump durability and helps in lowering the risk of pump thrombosis (Cheung et al., 2011; Maltais et al., 2018). While sternotomy‐sparing approach is preferable, as it is less invasive and decreases the risks of postoperative complications, for example, bleeding, thoracic wall instability etc., this approach requires the surgeon to be confident with apical coring and alternative outflow graft management, hence, it has a learning curve associated with it (Krabatsch et al., 2014; Maltais et al., 2014).

**FIGURE 3:**
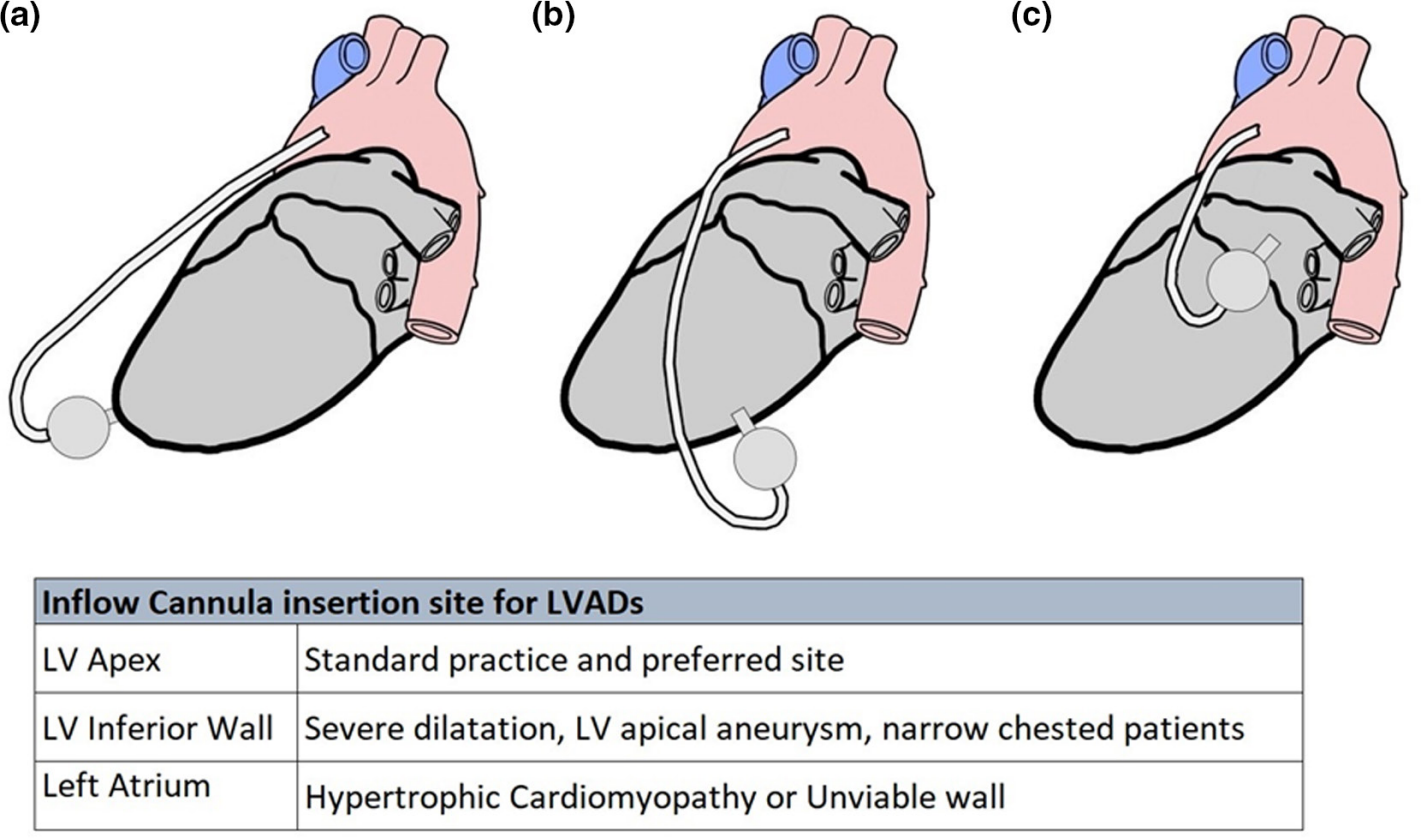
Illustration showing LVAD inflow cannula configurations from left lateral view. LVAD inflow cannula configurations. LVAD, left ventricular assist device

While the apex is favoured for LVAD inflow cannula insertion, anatomical complications such as apical aneurysm can restrict its use as a preferred insertion site. The inferior LV wall could be an alternative graft site when surgeons are challenged with a geometrically impaired dilated LV and apical aneurysm (Loforte et al., 2021; Potapov et al., 2019). In cases where challenged with hypertrophic cardiomyopathy, thin ventricle walls and/or LV wall calcification, left atrium (LA) is mandated as an inflow cannula graft site (Kiamanesh et al., 2020; Osaki et al., 2009). Figure 3 shows the inflow cannula insertion sites for the LVADs.

Anchoring method also appears to play an important role in the prevention of thrombus formation. An in vitro study tested four fixation methods, depending on whether “Core‐then‐Sew” or “Sew‐then‐Core” technique is used: (1) Epicardial (2) Transmural (3) Transmural with or (4) without back stitch. Epicardial stitching of the sewing ring showed the best outcome of pump stabilisation along with a reduction in risk of thrombus formation and bleeding. On the contrary, transmural without back stitch proved to be the least beneficial as the sutures were exposed to the blood through having a gap around the cannula which posed a high risk of thrombus formation (Hanke et al., 2017). Schmitto et al. utilised a less invasive approach and implanted HeartMate 3 via an upper hemi‐sternotomy combined with an anterior lateral thoracotomy approach. The pump was inserted into the left ventricle through an apical cuff and the outflow graft was anastomosed end‐to‐side to the ascending aorta (Schmitto et al., 2018).

The standard site for inserting the outflow cannula is the ascending aorta (Potapov et al., 2019). Using this insertion site requires accurate angling with appropriate prolene running sutures to allow for proper directional flow of the blood to reduce haemodynamic stress on the aortic root wall and aortic valve. Appropriate insertion practice reduces the incidence rates of aortic regurgitation (Bouabdallaoui et al., 2018). Having said that, the development of aortic insufficiency is one of the challenges faced after successful LVAD implants. Though it is manageable through concomitant aortic valve replacement, it poses a risk of recurrent heart failure and remains a clinical challenge to treat after an LVAD implantation (Bittner, 2018). A useful method to reduce LVAD‐related aortic insufficiency in patients with competent valves or even existing mild to moderate aortic insufficiency would be a simple coaptation stitch of the valve (Park et al., 2004). The ascending aorta should be avoided in case of heavy calcification, pseudoaneurysms or a hostile mediastinum, due to any previous surgery or mediastinum infection (El‐Sayed Ahmed et al., 2014; Potapov et al., 2019). Surgeons should pre‐operatively, decide upon alternative sites for outflow cannula placement, such as descending thoracic aorta, supra‐celiac abdominal aorta, brachiocephalic (innominate) artery, left axillary artery or subclavian arteries (El‐Sayed Ahmed et al., 2014; Kar et al., 2005). Table 1 lists some of the reasons for using different outflow graft cannulation sites.

**TABLE 1:** Outflow cannula insertion site for LVADs

Outflow cannula insertion sites	Reasons
Ascending Aorta	Standard practice and preferred site
Descending thoracic aorta	Unviable ascending aorta
	Cases of re‐entry (REDO) patients with hostile mediastinum
Supra‐celiac abdominal aorta	Unviable thoracic aorta
	REDO patients with hostile mediastinum
Innominate artery	For less invasive procedure
Axillary artery	Unviable thoracic aorta
	REDO patients with hostile mediastinum

**FIGURE 4:**
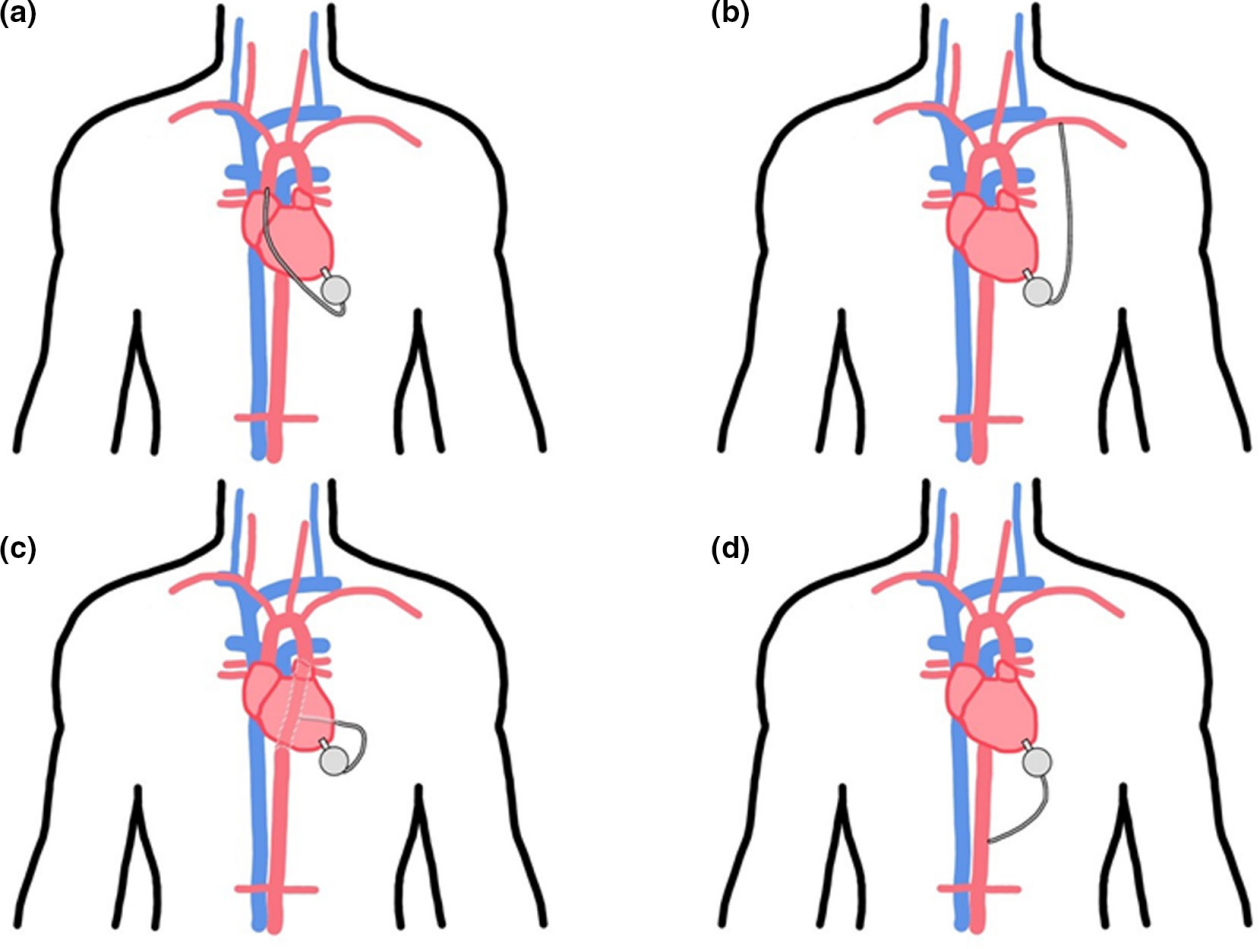
LVAD outflow cannula configurations. LVAD, left ventricular assist device

Various outflow cannulation sites are shown in Figure 4.

### 3.2 Stroke

Stroke is another relatively frequent complication that is associated with LVADs that increases the mortality risk. It has been established that 13%–30% of patient on LVADs experience a stroke (Han et al., 2018; Rogers et al., 2017). The eighth annual INTERMACS report reported that 18,591 LVAD devices had been implanted in patients in America by the end of 2016 (Kirklin et al., 2017). Both ischaemic and haemorrhagic stroke are implicated but the incidence of an ischaemic stroke is higher than a haemorrhagic stroke with an annual incidence of 5.5% versus 3.1%, respectively (Han et al., 2018). Thrombus deposition at the pump, aortic valve or on inflow or outflow grafts give rise to an ischaemic stroke while haemorrhagic stroke happens secondary to endocarditis, elevated blood pressure or haemorrhagic conversion of ischaemic infarcts (Han et al., 2018). MOMENTUM 3 trial established that a fully magnetically levitated continuous flow centrifugal pump (HeartMate 3) displayed a lower prevalence of stroke compared to continuous‐flow axial mechanical bearing pump (HeartMate II) at 2 years (10.1% vs. 19.2%, *p* = 0.02) (Mehra et al., 2018). This is a result of the enhanced design of the pump which reduces stasis by having an intrinsic pulsatility and also reduces shear stress due to wider blood‐flow paths thus significantly reducing the risk of pump thrombosis (Mehra et al., 2018).

### 3.3 Bleeding

Bleeding, mainly manifesting as gastrointestinal bleeding and epistaxis, is another complication following LVAD implantation (Han et al., 2018). The strict balance of the haematological system is disturbed when an LVAD is implanted due to the interaction between the blood products and the device surface leading to thrombosis or bleeding. Factors that increase the risk of bleeding with LVADs include impaired platelet aggregation, anticoagulation, low pulsatility and shear stress resulting in acquired von Willebrand syndrome, arteriovenous malformations and mucosal ischemia, particularly with gastrointestinal bleeding. 15%–30% of patients experienced gastrointestinal bleeding following LVAD implantation (Goldstein et al., 2015; Stulak et al., 2014). Management includes initiating proton pump inhibitors, administering blood products as necessary and reducing or temporarily discontinuing anticoagulation while closely monitoring as this would increase the risk of thrombosis (Han et al., 2018; Kilic et al., 2015).

### 3.4 Von Willebrand syndrome

Von Willebrand factor (vWF) is a protein involved in haemostasis which helps in platelet aggregation and sticking to damaged blood vessels to form a clot. VWF multimers bind to the collagen on damaged blood vessels, then platelets bind to the VWF causing platelet activation, adhesion, aggregation and thrombosis to prevent bleeding (Geisen et al., 2008). Acquired von Willebrand functional deficiency syndrome, specifically type 2A which is associated with LVAD devices, occurs as a result of the high local shear stress and blood flowing over the non‐biological surfaces of the device which increases platelet activation (Sisti et al., 2020). The increased shear stress causes structural changes in the shape of the vWF exposing the bonds between the amino acids thus leading to proteolysis of the highest molecular weight multimers of vWF which are the most active in haemostasis by the enzyme ADAMTS‐13 (Adatya & Bennett, 2015). Furthermore, the increased shear stress strengthens the effect of ADAMTS‐13 on VWF breakdown and increases its activity. This results in the reduction in high molecular weight VWF multimers in patients post‐LVAD implantation leading to impaired haemostasis and bleeding in 30%–75% of patients with continuous‐flow LVADs (Adatya & Bennett, 2015).

### 3.5 Pathophysiology of pump thrombogenesis

#### 3.5.1 Protein adsorption

Initial device contact immediately disrupts natural blood homeostasis, with the biomaterial surface immediately becoming coated with a thin boundary layer interphase of adsorbed plasma proteins, with a thickness in the order of 2–10 nm (Jaffer et al., 2015; Wilson et al., 2005). The rapid process (<1 s) of protein adsorption is widely regarded as the initiating event in thrombus formation since this protein layer is responsible for mediating many subsequent reactions (Jaffer et al., 2015; Mulzer & Brash, 1989; Santerre et al., 1992; Wilson et al., 2005). The dynamics of protein adsorption are related to the chemical and physical properties of the surface and the proteins (Jaffer et al., 2015; Lavery et al., 2017). Thus, adsorption involves interactions between charged groups at the protein–surface interface and/or conformational changes in protein structure. Surface adsorption is a reversible process, and the composition of the adsorbed proteins changes over time, a phenomenon is known as the Vroman effect (Jaffer et al., 2015). Pro‐thrombogenic plasma proteins, such as fibrinogen, vWF and fibronectin, are among the first proteins to deposit on the artificial surface, rapidly converting the implant into a biologically recognisable material (Biran & Pond, 2017).

#### 3.5.2 Platelet adhesion

Platelet activation may be elicited by a variety of mechanisms, including device‐related alteration in blood flow that triggers shear‐related platelet activation, and due to the adherence of circulating platelets to the adsorbed protein layer on the biomaterial surface, an event largely attributed to adsorption of fibrinogen (Biran & Pond, 2017; Lavery et al., 2017).

Fibrinogen is the primary protein responsible for platelet adhesion. The platelet‐fibrinogen interaction is mediated by the glycoprotein GPIIb/IIIa receptor, the most abundant integrin on the platelet surface. Ordinarily, GPIIb/IIIa receptors on circulating platelets must be conformationally activated via inside‐out signalling before it is capable of binding to adsorbed fibrinogen (Jaffer et al., 2015). While the exact mechanism remains elusive, GPIIb/IIIa receptors may detect and bind irreversibly to fibrinogen, even in their quiescent state (Jaffer et al., 2015, Lavery et al., 2017).

Once activated, platelets undergo a dramatic change in shape, triggering a series of complex biochemical pathways, culminating in the release of pro‐coagulant agonists such as adenosine diphosphate (ADP), thromboxane A2, calcium ions, p‐selectin and various coagulation factors (including coagulation factor V) from intracellular granules, which collectively amplify the cycle of platelet activation and attachment, and support cross‐talk with the coagulation, complement and immune systems (Lavery et al., 2017; Wiegner et al., 2016). Platelet aggregates formed through these diverse pathways associated with fibrin to form a platelet‐fibrin thrombus on the biomaterial surface, as shown in Figure 2 (Lavery et al., 2017).

#### 3.5.3 Activation of the coagulation cascade

Components of the blood contact system adsorb to the artificial surfaces of the LVAD, which facilitates the activation of the intrinsic pathway of coagulation, eventually culminating in the formation of fibrin. The interplay between platelets and the clotting cascade has also been identified wherein activated platelets release pro‐coagulant proteins (factor V) and intermediate clotting factors generated in the coagulation cascade are potent platelet activators (thrombin) (Abrams, 2006; Sukavaneshvar, 2017).

**FIGURE 5:**
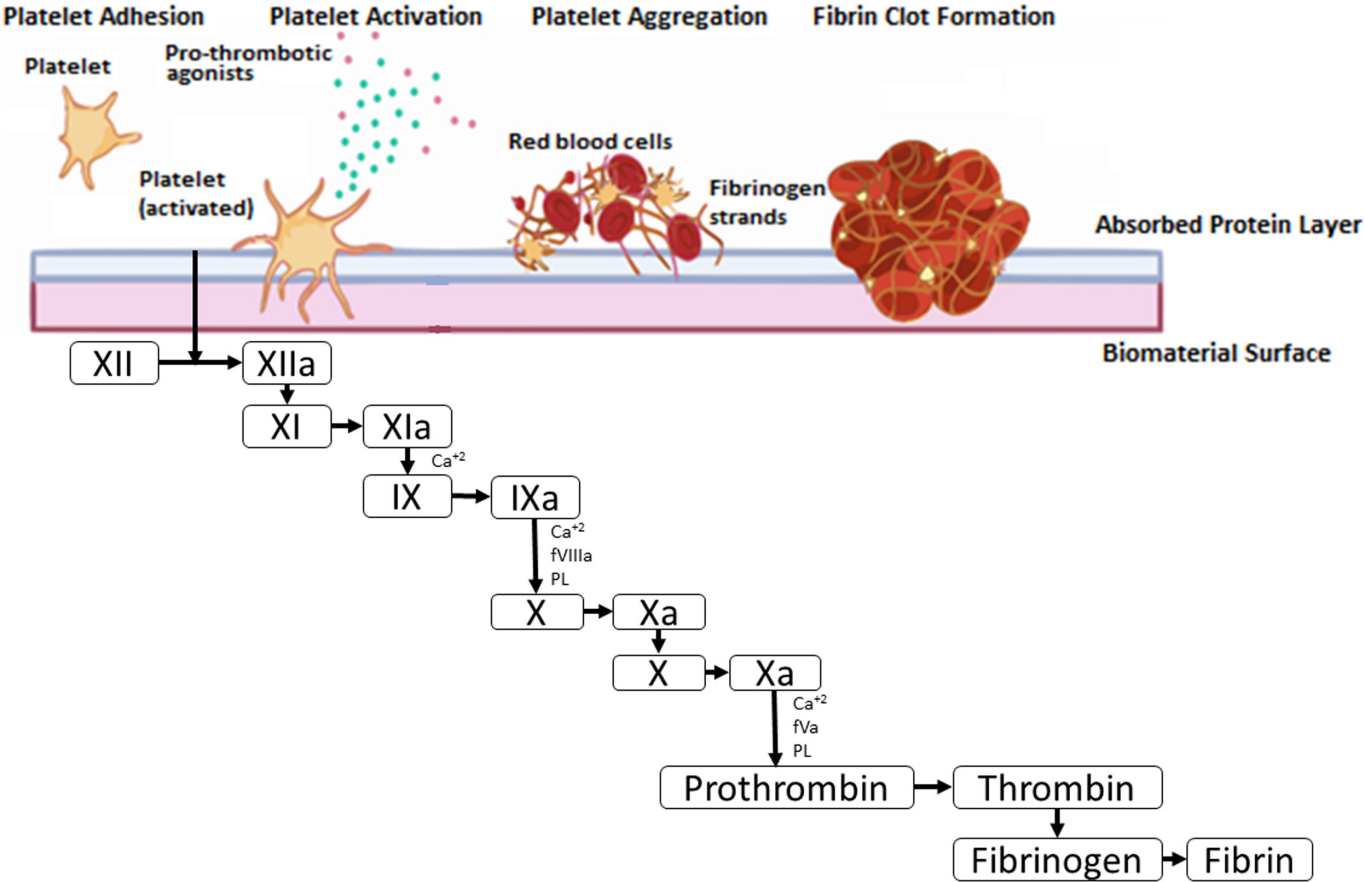
Simplified illustration of thrombus formation on biomaterial surface via activation of the coagulation cascade. Activation of the intrinsic pathway of coagulation culminates in the formation of fibrin

The biochemistry and molecular biology of these major processes have been studied extensively for decades (Sukavaneshvar, 2017, Ham Tran, 2006, Bates & Weitz, 2006). Figure 5 shows a simplified illustration of thrombus formation on the biomaterial surface via activation of the coagulation cascade. Recognition of the important role of the contact activation pathway to induce thrombus formation against biomaterial surfaces has led to attempts to use pharmaceutical agents inhibiting the initiation of the intrinsic blood coagulation cascade. As a result, fXII and fXI have emerged as promising targets for the prevention of thrombosis on blood‐contacting devices (Biran & Pond, 2017).

Anticoagulants such as heparin, rivaroxaban, and dabigatran and anti‐platelet agents such as aspirin, thienopyridines and glycoprotein IIb/IIIa inhibitors are widely used drugs for the prevention of thrombosis in many clinical settings (Biran & Pond, 2017; Sukavaneshvar, 2017).

#### 3.5.4 Anticoagulants used in patients with LVADs

Given the foreign nature of LVADs and its interaction with blood products which can affect the body's strict haemostatic control leading to thrombus formation and thus to increased morbidity and mortality in LVAD‐implanted patients, anticoagulation and antiplatelet therapy are warranted. Two types of clots have been found to occur in LVADs: (i) red clots, which are rich in red blood cells, and may be associated with blood stasis due to flow conditions or inadequate anticoagulation regimes, and (ii) white clots, which are rich in platelets with a fibrin mesh, that forms over time (Shah et al., 2017). Anticoagulation and the use of antiplatelet therapy are recommended for all patients implanted with an LVAD, however, the specific approach utilised differs across different mechanical circulatory support centres (Rimsans et al., 2016; Sage et al., 2018).

Warfarin, a vitamin K antagonist, is the main anticoagulant used post‐LVAD implantation. Warfarin is a coumarin anticoagulant which inhibits the vitamin K epoxide reductase complex 1 (VKORC1), an enzyme responsible for activating vitamin K, thus inhibiting the synthesis of vitamin K‐dependent coagulation factors II, VII, IX and X in addition to the anticoagulant proteins C and S (Patel et al., 2020). Warfarin does not have a direct effect on an established thrombus but it prevents thrombosis and embolism. Warfarin is dosed based on the results of the prothrombin time usually reported as the INR (International Normalised Ratio). The target INR for patients on warfarin therapy post‐LVAD implantation is 2–3 (Boehme et al., 2017). The time spent in this INR range is important to reduce the risk of thrombus formation or bleeding. A study conducted to examine the Proportion of Time spent in Target Range (PTTR) of INR 2–3 in patients with LVAD devices demonstrated that patients remained in the target range of INR 2–3 only 42.9% of the time indicating low anticoagulation control despite the rigorous anticoagulation and LVAD management (Boehme et al., 2017). Furthermore, compared to unfractionated and low molecular weight heparin, warfarin carries a lower thromboembolic risk (Rossi et al., 2012). As for antiplatelet therapy, aspirin is the main antiplatelet used at a dose of 81–325 mg/day alone or in combination with second antiplatelet dipyridamole or clopidogrel. Antiplatelets are used to prevent thrombus formation caused by the activation of platelets as a result of the shear stress and exposure of blood products to the artificial device surfaces (Jennings & Weeks, 2015). Combining oral anticoagulants with antiplatelet therapy increases the risk of bleeding and thus close monitoring is required.

**FIGURE 6:**
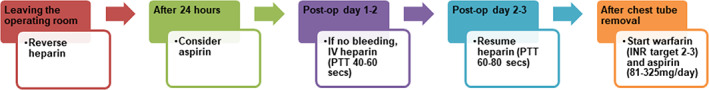
Recommended haemodynamic management for patients after receiving the axial‐flow HeartMate II LVAD. LVAD, left ventricular assist device

A ‘one size fits all’ approach cannot be applied to anticoagulant and antiplatelet management therapy with LVAD patients as different device types and pump flow have different indications and requirements in addition to individual patient factors which impact the response to anticoagulants and antiplatelets and thus the management approach. For example, early post‐operative anticoagulation management for HeartMate II, implantable centrifugal pumps and pulsatile MCS devices are primarily based on expert opinions published in 2013 that detail the recommended haemodynamic management in these patients and are summarised in Figure 6 (Feldman et al., 2013).

An individualised approach to therapy is required to balance the risk of thrombosis and bleeding (Adatya & Bennett, 2015). Essentially, it has been established that having an INR < 2 and a sub‐therapeutic dose of aspirin increases the risk of thrombosis but this should balance the risk of bleeding (Adatya & Bennett, 2015).

## 4 MANAGEMENT OF LVAD PUMP THROMBOSIS

**FIGURE 7:**
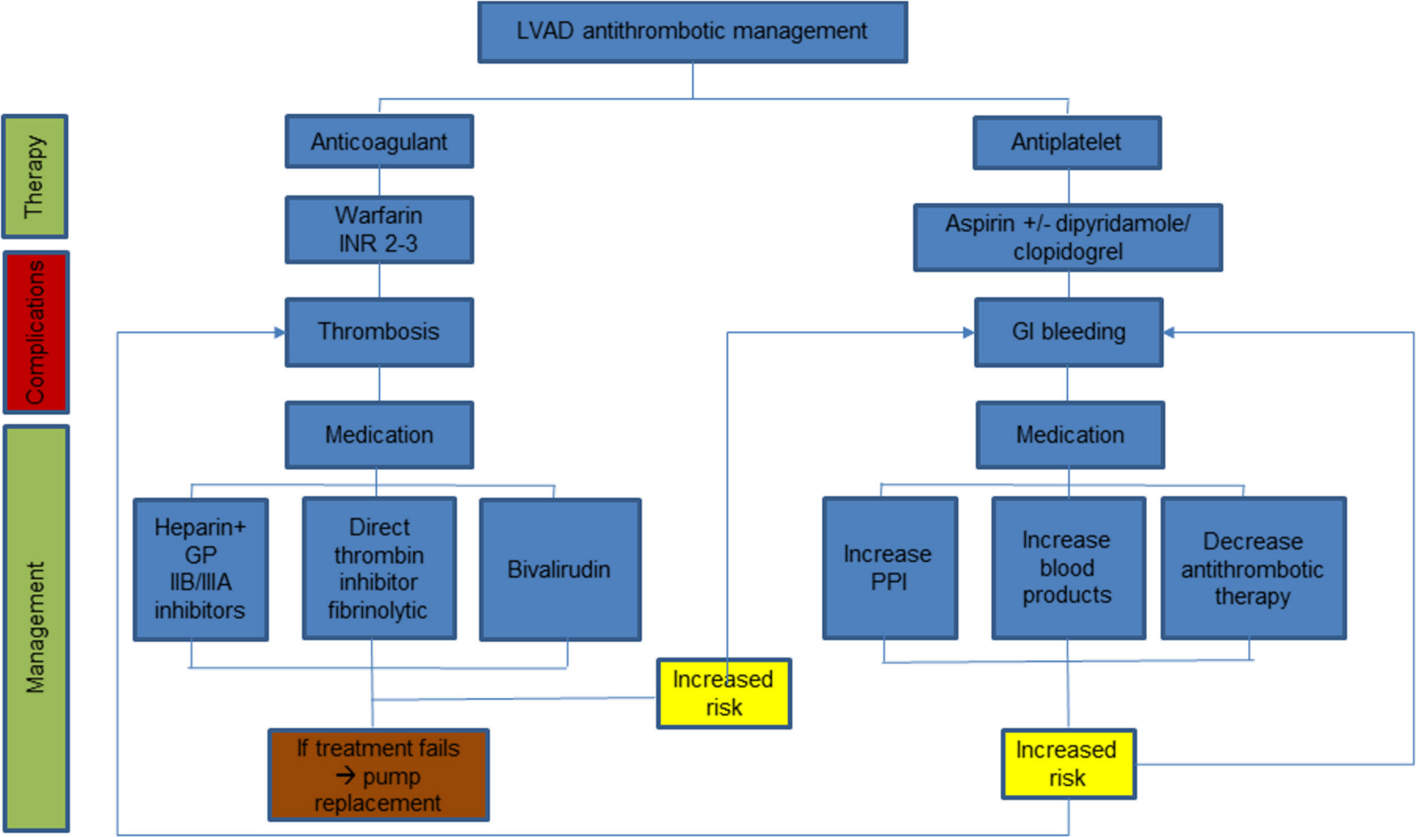
Summary of anti‐thrombotic management for left ventricular assist devices. GI, gastrointestinal; GP, glycoprotein; LVAD, left ventricular assist device; PPI, proton pump inhibitor

As mentioned earlier, pump thrombosis is one of the main complications that arise post‐LVAD implantation that affects morbidity and mortality and can lead to the requirement for either LVAD device replacement or explantation. Elevated lactate dehydrogenase can occur up to 3 months prior to pump thrombosis which can be monitored routinely as a biomarker for haemolysis and pump thrombosis early detection and thus management (Rogers et al., 2017). Pump thrombosis can be caused by both a red thrombi which is a fibrin mesh with trapped red blood cells that develops rapidly or a white thrombi which is aggregated platelets and debris that develops slowly over time. Since they are composed of different components, thrombolytic treatment would be successful with a red thrombus while white thrombus would respond better to glycoprotein (GP) IIB/IIIA inhibitors (Adatya & Bennett, 2015). Pump thrombosis is managed in numerous ways, which include increasing the intensity of anticoagulants and antiplatelets; initiating antiplatelet therapy; initiating thrombolytic agents like fibrinolytics, for example alteplase (tPA) and glycoprotein (GP) IIB/IIIA inhibitors, for example eptifibatide, antithrombotics, pump replacement or explantation (Rimsans et al., 2016; Rogers et al., 2017). These practises are summarised in Figure 7.

In 2014, Najjar et al. analysed the incidence of pump thrombus in the 382 patients who received an HVAD under the BTT and CAP protocols (Levine & Gass, 2019; Najjar et al., 2014). The effectiveness of various treatment strategies and predisposing factors to the development of pump thrombus was also evaluated (Najjar et al., 2014). Three hundred eighty‐two patients were involved in the study who had the HVAD device implanted, of these 8.1% experienced pump thrombosis and 4.2% required pump replacement (Najjar et al., 2014). Patients were successfully managed with medical therapy in 50% of thrombus case, which consisted of heparin, glycoprotein (GP) IIB/IIIA inhibitors, for example eptifibatide and alteplase (tPA) used alone or in combination. It was found that alteplase (tPA) successfully treated pump thrombosis in 63% of patients, eptifibatide used alone or in combination with heparin succeeded in 50% of patients and the use of heparin alone was not successful (Najjar et al., 2014). While these findings provide support for the role of medical therapy in managing pump thrombosis, the small number of thrombosis events and the heterogeneity of therapies used make it difficult to make strong inferences about preferred strategies for the management of pump thrombosis (Najjar et al., 2014). Additional studies are needed to gain a deeper understanding of the preferred regimen, dosage and duration of medications that will provide the optimal balance between efficacy and safety. Multivariable analysis revealed various risk factors for pump thrombus, including sub‐therapeutic international normalised ratio (INR > 2), sub‐optimal anticoagulation and antiplatelet therapy‐elevated mean arterial blood pressure < 90 mmHg (Najjar et al., 2014).

Bivalirudin is an alternative anticoagulant to heparin that is administered intravenously which interacts with both free and clot bound thrombin and reversibly inhibits it thus inhibiting the activation of factors V, VIII, XIII and protein C and is less immunogenic compared to heparin thus avoiding heparin‐induced thrombocytopenia (HIT) (Adatya & Bennett, 2015; Rimsans et al., 2016). An algorithm for the diagnosis and management of suspected pump thrombosis constructed by a multidisciplinary team and published by the international society for heart and lung transplantation working group detailing the proposed algorithm according to the clinical presentation of the patient has been developed as a potential guide that can be adhered to in order to manage pump thrombosis in patients (Goldstein et al., 2013).

It should be noted that pharmacological management of pump thrombosis is associated with a high proportion of treatment failure eventually leading to the need for pump replacement (Adatya & Bennett, 2015). Pump replacement is required in patients with obvious pump thrombosis, pump blockage and in shock, unresponsive to battery and controller exchanges without further diagnostic studies (Goldstein et al., 2013).

## 5 COATING MATERIALS FOR MECHANICAL CIRCULATORY SUPPORT DEVICES TO MITIGATE BLOOD COAGULOPATHY

Modification of medical device surfaces to improve blood compatibility has been pursued to reduce device‐related thrombus formation and inflammatory reactions. Surface modification technologies can be assigned into two broad categories: (a) passivation of material surfaces and (b) bioactive surface treatments and coatings (Biran & Pond, 2017; Tanzi, 2005). Passive approaches are aimed at reducing the inherent thrombogenicity of the material surface by establishing a barrier at the device‐blood interface through modification of surface chemistry (e.g. hydrophilicity) or material physical structure (e.g. topography). Bioactive strategies employ direct pharmacological inhibition of the coagulation response by local drug delivery or permanent immobilisation of an active agent (Sin et al., 2012; Tanzi, 2005).

### 5.1 Passive coatings

#### 5.1.1 Diamond‐like carbon

Diamond‐Like Carbon (DLC) Coating is currently the most commonly used passive coating, being used on devices such as the VentrAssist LVAD (Sin et al., 2012, Meer et al., 2003). This coating is formed from a metastable form of amorphous carbon, which contains a significant amount of tetrahedral $sp^{3}$ hybridised atoms, along with varying amounts of hydrogen (Wagner et al., 2020). DCL films and coatings may be easily deposited on a wide variety of substrates, through chemical vapour deposition (CVD) and physical vapour deposition (PVD), which have been reviewed in detail elsewhere (Fedel, 2013). DLC coatings are characterised by their excellent mechanical, chemical and electrical properties, making them most appealing for biomedical applications. When compared to other blood‐interfacing materials, such as titanium alloy, polycrystalline diamond and expanded polytetrafluroethylene (ePTFE) (Jones et al., 2000; Schaub et al., 2000; Sin et al., 2012). DLC‐coated surfaces demonstrate low levels of interaction with the immune system, clotting factors and platelets due to their hydrophobic nature, chemical inertness and low frictional coefficient (Jones et al., 2000; Zeng et al., 2016). As a result, DLC coatings may be applied to reduce the incidence of thrombosis.

A DLC coating was tested in vivo with the EVAHEART centrifugal VAD (SunMedical Technology, Japan), maintaining a stable performance without any adverse effect over 6 months in calves under the postoperative anticoagulation protocol. Blood‐contacting surfaces of the DLC coating were entirely free from thrombus formation. The wear rate of the seal face was 10 μm after 200 days of continuous operation, with no measurable wear at the hydrodynamic bearing (Wagner et al., 2020; Yamazaki et al., 1998).

One of the major limitations of this coating is the possible formation of micro‐cracks on its surface, leading to increased internal stresses, followed by subsequent film breakdown (Zeng et al., 2016). If adhesion is insufficient, the coating may spontaneously delaminate as a result (Zeng et al., 2016). To counter this, modified DLC coatings with elastic features are being researched (Shirakura et al., 2006; Sin et al., 2012).

#### 5.1.2 2‐methacryloyloxyethyl phosphorylcholine

Coating surfaces with zwitterionic films is an effective method of enhancing the biocompatibility of an implantable device (Schlenoff, 2014). Phosphatidylcholine, the major phospholipid found on the outer surface of the mammalian cell membrane resists cell and protein adhesion due to its zwitterionic polar head group being electronically neutral at physiological pH. 2‐methacryloyloxyethyl phosphorylcholine (MPC) polymer incorporates the phosphatidylcholine head group within its polymer backbone, and thus, effectively suppresses thrombin formation and the associated adsorption and activation of platelets, even in the absence of anticoagulants, rendering it as anti‐thrombogenic (Ishihara, 2019; Jaffer et al., 2015). The EVAHEART® LVAD consists of a titanium pump, modified with a coating of MPC polymer on all blood‐contacting surfaces in order to maximise hemocompatibility.

However, the MPC polymer has been found to be biodegradable, and as a result, the anti‐thrombogenic properties of MPC‐coated material may possess a limited lifetime, meaning the patient will eventually require antithrombotic therapy (Sin et al., 2012). A recent study by Kihara et al. found that after 45 days (±32 days), only 60% (±37.2%) of the pump surface was still coated with MPC (Kenji et al., 2004).

#### 5.1.3 Textured surfaces

Biomaterial surfaces serve as critical interfaces between the host and implant to coordinate many biologic processes (Wagner et al., 2020). Historically, medical devices that incorporate textured blood‐contacting surfaces have shown greater biocompatibility than smooth constructs. This is because the textured surfaces promote the adhesion of circulating endothelial cells to form a stable ‘neointimal’ lining, which minimises thromboembolic risk and reduces the level of required anticoagulation therapy (Wagner et al., 2020). Similarly, the textured surface increases the constructed surface area, creating space for cells to attach and proliferate (Wagner et al., 2020).

Examples of textured topographies include nano‐dimples, nanofibers and microgratings. A variety of manufacturing technologies exist that allow for controlled textured surfaces, at the nano‐ and micro‐scale, including solid free‐form machining, moulding, stereolithography, laser machining and sintering (Bose et al., 2018). The HeartMate family of LVADs utilise inflow cannulae which are fully textured with sintered titanium microspheres, along with textured polyurethane linings. Textured polyurethane membranes also reduce the adhesion of the pathogen Staphylococcus aureus, thereby reducing the risk of device infections (Bose et al., 2018; Noviani et al., 2016; Shah et al., 2017).

It is important to note that not all textured surfaces lead to the production of a neo‐intimal layer formed exclusively from endothelial cells. In 1993, Salih et al reported the presence of at least two different cell populations on the textured surfaces of ThermoCardiosystems LVADs (Salih et al., 1993). Therefore, endothelial progenitor cell deposition onto the blood‐contacting surfaces may be considered to induce or trigger the formation of an endothelial layer. Alternatively, textured surfaces may also be seeded with endothelial cells, followed by in vitro culture prior to implantation, which will be explored in the later sections (Sukavaneshvar, 2017).

### 5.2 Active coatings

One strategy to address the challenge of pump thrombosis using anti‐thrombogenic drugs without increasing bleeding risk is the surface modification of the device biomaterial surface with anti‐thrombogenic drug molecules (Jordan & Chaikof, 2007; Sukavaneshvar, 2017). The aim of this approach is to localise the effect of the anti‐thrombogenic drug around the device, without systemic suppression of necessary hemostatic function (Sukavaneshvar, 2017).

#### 5.2.1 Heparin

Heparinization is the most popular technique for device surface modification due to its potent anticoagulant properties (Biran & Pond, 2017). Depending on its molecular weight and structure, heparin binds to antithrombin‐III which is present in the blood plasma, significantly increasing the potency of antithrombin‐III's to inactivate factor Xa and thrombin that may be generated on the device surface (Biran & Pond, 2017). Since these clotting factors are key mediators of thrombosis, their inactivation limits device thrombosis. Moreover, since thrombin is also a potent platelet activator, the presence of heparin‐binding heparin into an inactive complex should help minimise platelet activation, leading to improve hemocompatibility of the implant material (Wagner et al., 2020).

Investigators have been able to covalently, ionically and physically attach heparin to various substrates utilising a number of chemistries.

#### 5.2.2 Ionically bound heparin

Ionic approaches involve binding the highly negatively charged heparin onto a cationic surface through ionic binding, with heparin being slowly released into the bloodstream. However, a serious limitation of ionic bonding is the risk of leaching of heparin, which will eventually leave the surface unprotected (Wagner et al., 2020).

#### 5.2.3 Covalently bound heparin

To impart a degree of activity longer than that possible with ionic linkages, covalent chemistries were developed to immobilise heparin onto the surface (Wagner et al., 2020). While several variations of surface immobilised heparin have been developed, which utilise diverse linker chemistries it is now recognised that the conformation of the attached heparin and the point of attachment (end point vs. multipoint) are critical factors determining the catalytic efficiency of the immobilised heparin (Sukavaneshvar, 2017). Some of the notable examples of commercial coatings making use of covalent bonding are Carmeda BioActive Surface (CBAS) from Medtronic, which is used on devices such as the Berlin Heart INCOR LVAD, and Trillium, developed by BioInteractions Ltd.

Carmeda BioActive Surface consists of depolymerized, lower molecular weight heparin covalently grafted to biomaterial surfaces by aminating the base matrix, which consists of positively charged polyethyleneimine and negatively charged dextran sulphate deposited in a layer‐by‐layer fashion. This method was developed by Larm et al (Larm et al., 1983). What is unique to Carmeda Bioactive Surface is that it retains the functional activity of the immobilised heparin, specifically its capacity to bind to the coagulation inhibitor anti‐thrombin, present in the bloodstream (Hosseinipour et al., 2017; Larm et al., 1983). Inactive complexes formed on the immobilised heparin are released and swept away by the blood flow and excreted. As a result, covalently bound heparin is not consumed in the reaction but remains active and available for further inhibition.

Trillium, developed by BioInteractions Ltd. and marketed by Medtronic, is a tri‐functional polymer coating, which provides endothelial‐like characteristics to the surface of the VAD. A priming layer is first bound to the blood‐contacting surface, followed by the deposition of a hydrophilic functional layer with covalently bound heparin (Hosseinipour et al., 2017). Sulphate and sulfonate groups, which are incorporated into the functional layer emulate the negative charge of the vascular endothelium. These negatively charged polymers repel negatively charged platelets, preventing platelet adhesion and activation. Polyethylene oxide polymer, which is present within the functional layer is hydrophilic in nature, thereby creating an insulating water layer between the blood and artificial surface that resists cell adhesion and protein deposition (Biran & Pond, 2017; Hosseinipour et al., 2017).

#### 5.2.4 Limitations of heparin‐binding

Despite these promising attributes, LVADs coated with heparin have not been associated with significant reductions in thromboembolic events. This is due to the fact that heparin is only an anticoagulant and not an antiplatelet agent (Sukavaneshvar, 2017). This limitation is particularly relevant in LVADs, where platelets play a major role in thrombus development, that is where high shear and disturbed flow lead to platelet activation and aggregation even in the presence of anticoagulants (Sukavaneshvar, 2017). Moreover, due to its biodegradable nature, heparin coatings have a finite lifespan, which is in contrast to DLC coatings. As a result, this drug‐device combination has not eliminated the need for systemic anti‐thrombogenic drug usage to minimise the risk of pump thrombosis. Considering the multifactorial nature of device thrombosis, it is not surprising that strategies aimed at inhibiting one step of the thrombotic process have not succeeded entirely and hybrid concepts that incorporate two or more anti‐thrombogenic properties are being increasingly explored (Lih et al., 2016; Wang et al., 2013).

#### 5.2.5 Endothelial cell lining

The endothelial cell presents and releases a number of biologically active agents that limits thrombogenesis in vivo. As such, approaches to mimic these properties, such as seeding or otherwise reconstituting endothelial cells on the surface of the LVAD are been actively pursued as a method of enhancing biocompatibility (Sukavaneshvar, 2017). The use of endothelial cell linings remains controversial as it does not work in areas of pump with small clearances. These surfaces may also partially lose function over time, or may wash away with blood flow. Extraction of the epithelial cells and texturing of the surfaces is difficult, creating further challenges these coatings. They also offer no guarantee of function, as cells may wash away by the blood upon implantation (Sin et al., 2012).

Each different surface coating comes with its own benefits and its own challenges. The current challenge is to produce an MCS device with an optimal design and coating combinations as to offset downfalls and compound benefits.

## 6 DEVICE DESIGN IMPROVEMENT TO MITIGATE LVAD‐RELATED BLOOD COAGULOPATHY

The HeartAssist5 is the modern version of DeBakey/NASA VAD that has been under development since 1988 (Agarwal & High, 2012; Hosseinipour et al., 2017). A unique feature of the HeartAssist 5 device is the addition of an integrated ultrasonic flow probe around the outflow graft that collects real‐time measurements of blood flow as it moves from the outflow graft into the aorta (Hosseinipour et al., 2017). Pump parameters, including pump flow (L/min), power consumption (watts) and pump speed (rpm), are also recorded, which provides the physician with real‐time information on the patient's cardiac activity, and will alert them in instances of unusual activity. Extensive work has been carried out to refine the pump geometry in order to maximise hemocompatibility. Through careful study of the system's computational fluid dynamics, much optimisation has been done on refining the internal geometry of the pump, such that blood is drawn smoothly through the LVAD resulting in less platelet activation and hemolysis. The impeller, flow straightener and diffuser are designed such that they eliminate any area of blood stagnation, thereby preventing thrombus formation. All blood‐contacting surfaces are made of highly polished titanium with Carmeda BioActive Surface coating, which is known for its excellent biocompatibility (Hosseinipour et al., 2017).

In line with other third‐generation LVADs, the DuraHeart LVAD incorporates a centrifugal flow rotary pump with a magnetically suspended impeller to provide long‐term durability (Agarwal & High, 2012). The system is also supported by a secondary hydrodynamic bearing in the event of the magnetic system failing. What makes the DuraHeart unique is the utilisation of three electromagnetic coils and position sensors to precisely control the impeller's position within the pump housing (Agarwal & High, 2012). The large gaps between the impeller and the blood chamber walls (250 microns on each side) enable improved washout, preventing thrombus formation inside the pump chamber. These gaps also translate into reduced shear stress and a corresponding hemolysis reduction. The pump's blood‐contacting surfaces are made of both titanium and stainless steel, with an additional coating of stable covalently bound heparin to enhance blood compatibility and reduce the risk of thrombus formation in low‐flow areas (Agarwal & High, 2012).

The HVAD pump was also revolutionary in its application of magnetic suspension and hydrodynamic thrust bearing technology to support the impeller within the pump housing (Chatterjee et al., 2018). Briefly, the impeller is passively suspended by magnets located within the pump, while hydrodynamic thrust bearings which are located on the four wide‐channel impeller blades provide the necessary axial forces to balance the impeller's position within the pump housing (Chatterjee et al., 2018). Its wide‐blade impeller features three blood flow paths, which are designed to enhance blood flow and reduce blood trauma while reducing the blood travel time through the device (Chatterjee et al., 2018). The device possesses an integrated inflow cannula which minimises the pump footprint within the chest cavity, allowing the pump body to be implanted entirely within the pericardial space, eliminating the need for a pump pocket. The pump housing is composed of titanium and the thermoplastic polyester ether ketone, for improved durability (Agarwal & High, 2012).

The most recent LVAD introduced for clinical use is the HeartMate 3. This third‐generation pump was designed with a focus on mitigating the risk of thrombosis as seen with its predecessor, the HeartMate II (Hosseinipour et al., 2017; Levine & Gass, 2019). The centrifugal pump utilises full magnetic levitation to maintain its impeller within the blood path, with wide blood flow passages to reduce shear stress. This updated design is also pre‐programmed with speed modulation technology, which uses rapid changes in rotor speed every 3 seconds to mimic an intrinsic, physiological pulse (Levine & Gass, 2019). The pump is composed of sintered titanium microspheres, which establish an endothelial tissue interface at the boundary of the device.

## 7 ROLE OF SHAPE MEMORY ALLOY MATERIALS IN ANCHORING OF LVAD

As we are moving towards less invasive techniques of LVADs implantation, the anchoring mechanism plays a crucial role in the stabilisation of the device and prevention of thrombus formation. Future holds for miniaturised LVADs that may be delivered via a transcatheter delivery system, without giving any incision on the body. Shape memory alloys have been a burgeoning technological field. Their benefit in medical and other commercial applications lies in their mechanical characteristics, particularly their shape memory abilities. There are many iron and copper‐based alloy combinations available, however, nickel‐titanium (NiTi) alloys are most often used due to better stability, biocompatibility and performance (Jaronie Mohd Jani et al., 2014, Morgan, 2004). These contain a composition of approximately 50% of both nickel and titanium. NiTi has been used in widespread industrial markets since its development and began to be used in medical devices in the 1980s (Levi et al., 2008). While nickel is a common allergen, and titanium has historically been used in medical implants, in combination, as nitinol, these have been found to be biological inert (Levi et al., 2008; Morgan, 2004). This is an important feature of the materials as, throughout their functional period, they need to have little to no effect on the biological system in which they operate, to avoid any allergic reactions or corrosion leading to the release of ions into the bloodstream (Jaronie Mohd Jani et al., 2014, Machado & Savi, 2003). This allows them to have potential in the delivery, long and short‐term use, and removal of devices.

The properties that define shape memory alloys are their ability to be manipulated in and out of their original shape and/or size, as triggered by various influences such as temperature or magnetism (Jaronie Mohd Jani et al., 2014). Nitinol, in particular, can easily be made compact through compression, in order to fit into even smaller dimensions than similarly elastic materials. Furthermore, it has the elastic properties to reorganise into its functional design (Levi et al., 2008). This can be designed as an automated process as the materials are ‘trained’ to undertake certain forms under certain conditions by an external force. Through this property they have found the most use as actuators in medical devices, acting as the component primarily responsible for the support and control of the device's deployment and action. (Jaronie Mohd Jani et al., 2014) The nitinol material is set into its desired shape (a ‘trained’ formation) by applying a crystallisation temperature of approximately 500°C and then rapid cooling (Levi et al., 2008). At low temperatures, nitinol takes on the martensite phase and is very malleable, as the low‐temperature results in a low‐energy, symmetrical border configuration (Aaronson et al., 2012). In these conditions, the material is condensed into the size and form necessary for its implantation. Once in conditions of increased temperature, such as when heated to internal body temperature upon implantation, the structure transforms to the austenite state, which, as a cubic structure, maintains the shape's stability in the trained formation (Levi et al., 2008; Machado & Savi, 2003). It will be maintained in the austenite state as long as the environment remains stable as it can be reversed into the martensite phase via low temperature or stress. This can include the stress placed on it by the work of the system that the material is placed in, which results in a strain on the material. A significant advantage of the shape memory designs is that they achieve an optimal stress–strain balance in the austenite state, similar to that of human bone and tissue (Morgan, 2004). This involves resisting compression over a range of forces (that it may experience in a biological system) and recovering from them into their original shapes.

The shape memory effect characteristics are what make this resistance possible. Pseudo‐elasticity, also known as super‐elasticity, is a shape memory effect characteristic demonstrated in alloys when heated above the temperature required to reach the austenite state (Jaronie Mohd Jani et al., 2014; Machado & Savi, 2003). Super elasticity is best achieved when the composition of the nitinol has a slightly higher nickel content (Hord et al., 2019; Morgan, 2004). It allows the nitinol to take on a shape appropriate to the load, or stress, placed upon it when changes occur in the system—such as in the changing diameter of a cardiac chamber during contraction. The two‐way shape memory effect outlines the mechanism through which the material returns to its trained shape, without mechanical loading (Machado & Savi, 2003). Instead, the phase transformations occur due to temperature changes, and the shapes formed after cooling and heating are distinct. For the material to exhibit this characteristic it requires cyclic training wherein the material is modified in and out of the martensite and austenite phases under a variety of loads, in order to avoid stress‐induced phase transformations (Machado & Savi, 2003). However, this process does result in a less flexible re‐arrangement of the crystalline organisation—known as plastic strain—which has an irreversible effect on the structure. The one‐way shape memory effect occurs when the material is shaped while in its cool, martensite phase by an external force. This stress placed by this force temporarily changes the materials crystal structure. It can then be reverted back to its original form if it is heated—known as a reverse phase transformation (Machado & Savi, 2003).

A current widespread application of nitinol has been in the use of ‘self‐expanding’ stents wherein they are compressed into sheaths at cold temperatures, deployed via catheter and expanded once heated to body temperature and their sheath is removed (Levi et al., 2008). Further, their use in cardiac support devices is still being explored with extensive possibilities. Cardiac compression devices have seen some of the greatest potential. The ParaCor HeartNet is a biventricular mesh restraint device comprised of nitinol that is designed to provide flexible support to a dilated heart, that easily conforms to its size and shape (Varela et al., 2019). The advancements in the utilisation of such materials in cardiovascular devices look promising, as they have huge potential to be used as an anchoring system for an MCS device like an LVAD and eventually an incisionless procedure, for example, via transcatheter delivery route. As described above in Section 3.1.1.3, current implantation requires either sternotomy or sternotomy‐sparing procedures like left lateral thoracotomy. The LVAD is usually secured using sutures, however, with a transcatheter delivery technique, nitinol can be used to anchor an LVAD from inside the left ventricle. Furthermore, if device removal is required, instead of surgical removal, their shape memory feature may allow the surgeons to remove the device via a transcatheter system.

## 8 CHALLENGES IN PRE‐CLINICAL TESTING OF MECHANICAL CIRCULATORY SUPPORT DEVICE PUMP THROMBOGENICITY

The International Organisation for Standardisation (ISO) defines guidelines pertaining to the evaluation of medical devices, derived from multiple national and international standards. In our evaluation of MCS devices, it is important to acknowledge that these guidelines for in vitro thrombogenicity tests differ depending on the device being assessed. In vitro studies can be subdivided into either static models or dynamic models, with both subtypes coming with their own inherent coagulopathic advantages and disadvantage (Sarode & Roy, 2019). Static models are preferred for studying the interaction between blood and material but are limited due to a lack of actual blood flow. Dynamic models allow for a more complete analysis of thrombosis with blood flow, however, they are highly unstandardized (Sarode & Roy, 2019). These inherent differences pose a challenge in the comparison and evaluation of MCS devices. There is a lack of guidance on the use of testable markers for thrombosis in static and dynamic models, and as a result, most thrombosis testing occurs in vivo. While in vitro thrombosis testing does occur, it tends to be unstandardized. Most in vitro testing of MCS devices only measures haemolysis, and the focus of these tests tends to be on the MCS devices operational performance, as opposed to a thorough evaluation of coagulopathy (Sarode & Roy, 2019). A thorough examination would study thrombosis, haemolysis, vWF levels, coagulation factor levels, etc. In vitro studies also require the use of an anticoagulant, which may not always be used in a clinical setting (Sarode & Roy, 2019). Although the ISO guidelines are not intended to provide a rigid set of standards, there is a need for clearer guidance and risk‐management perspectives when it comes to the selection of materials and in vitro screening methods in the development of MCS devices (Bernard et al., 2018).

Many pro‐thrombotic factors generated using in vivo studies are cleared from the blood and hence, detectable, giving this model an advantage over in vitro study. However, in vivo studies also come with their own pitfalls. They are expensive, time consuming and thrombotic markers can only be tested during symptomatic phases of the disease, leaving no room for asymptomatic intervention (Sarode & Roy, 2019). While these studies can be useful, the results do not always translate appropriately to humans due to physiological differences between species (Sarode & Roy, 2019). Hence, there is a need for a robust high‐fidelity in vitro or ex vivo testbed to test various aspects of intracardiac cardiac devices during their developmental phase. Most recently, Park et al developed an organosynthetic dynamic heart model whereby preserving the intracardiac structures and replicating the left ventricular motion using soft robotics (Park et al., 2020). These advancements are crucial for the development of novel intracardiac devices.

It is also important to note that the material used in an MCS device is not the sole factor in MCS device suitability, and advancements of MCS devices cannot be taken in isolation. While the biocompatibility of the device is of high importance, we must consider the material as a component of an MCS device, as opposed to a separate entity. It is possible that the best choice of material with regards to its biocompatibility may lessen the functionality of the MCS device itself. Factors such as size, durability and shear stress produced as a result of the machines function affect the suitability of a device for a given patient. Coupling this knowledge with the known co‐morbidities associated with patients requiring an MCS device, it is clear that the decision to place a patient on a particular MCS device is multifactorial, and as such should be decided on a case by case basis.

With regards to pump thrombosis, risk factors can be sub‐divided into patient, device and management‐related factors. Patients requiring MCS devices tend to be suffering from advanced‐stage heart failure, and are therefore more susceptible to a pro‐thrombotic event (Kilic et al., 2015). Patients with end‐stage heart failure are also more prone to developing thrombi in areas of pre‐existing scar tissue of the left ventricle (John et al., 2008). With device‐related pump thrombosis, factors such as local heat generated by the pump and outflow graft kinking are at play. Post‐operative management with appropriate anticoagulants may decrease the risk of pump thrombosis, and thus improve patient outcome. However, in cases where patients develop a bleeding event, it may become necessary to reduce or discontinue the use of anticoagulants given that the bleeding risks are exacerbated with anticoagulation, and this raises the risk of pump thrombosis (John et al., 2008).

It is, therefore, evident that even with the advancements being made in MCS devices, like an LVAD, there is still a place for antithrombotic therapies. When treating patients that require an MCS device, a balance must be struck so as to avoid complications of either bleeding or thrombosis.

## 9 CONCLUSION

MCS devices, such as LVADs, have revolutionized the treatment of end‐stage heart failure over the last three decades. Advances in biomedical engineering have resulted in significant size reductions, optimised pump efficiency and enhanced clinical applicability, thereby offering an effective long‐term therapeutic option for the management of patients who are both eligible and ineligible for heart transplantation.

While their benefits are indeed significant, pump thrombosis remains the Achilles heel of LVADs. Current treatments for pump thrombosis consist of aggressive anticoagulation and antiplatelet therapies, however, these therapies may be associated with increased risks of gastrointestinal bleeding. In cases of severe pump thrombosis, device exchange or urgent transplantation may be required. Due to the multifaceted nature of pump thrombosis, a ‘one size fits all’ approach is not appropriate. An extended personalised approach that incorporates the patient's unique hemostatic conditions is needed to balance the risk of bleeding and thrombotic complications. As such, understanding the predisposing risk factors for a pump thrombosis is critical to devising preventive strategies. Despite these obstacles, significant progress has been made in methods to mitigate device thrombosis, particularly in the design of pump geometry and surface modifications for blood‐contacting devices. Improved materials, combined with refined, personalised drug regimens have reduced the risk of thrombosis, rendering many devices clinically acceptable for a vast majority of patients.

Going forward, future MCS devices must focus their attention on hemocompatibility and on individualised anticoagulation targets, which are crucial for mitigating the occurrence of device thrombosis and improving the rates of overall patient survival. The lack of robust guidelines for the management strategy of pump thrombosis by pharmacotherapy warrants further research in this area.

## DISCLOSURES

The authors declare that they have no conflict of interest.

## Data Availability

Data sharing is not applicable to this article as no datasets were generated or analysed during the current study.
